# Biomechanical considerations in the pathogenesis of osteoarthritis of the elbow

**DOI:** 10.1007/s00167-015-3518-7

**Published:** 2015-02-13

**Authors:** Andras Heijink, Matthias Vanhees, Kimberly van den Ende, Michel P. van den Bekerom, Roger P. van Riet, C. Niek Van Dijk, Denise Eygendaal

**Affiliations:** 1 Department of Orthopaedic Surgery, Academic Medical Center (AMC) Amsterdam, Meibergdreef 9, 1105 AZ Amsterdam, The Netherlands; 2 Department of Orthopaedic Surgery, AZ Monica, Antwerp, Belgium; 3 Department of Orthopaedic Surgery, Amphia Hospital, Breda, The Netherlands; 4 Department of Orthopaedic Surgery, Onze Lieve Vrouwe Gasthuis, Amsterdam, The Netherlands

**Keywords:** Cartilage, Post-traumatic, Osteochondritis dissecans, Instability, Malalignment, Aetiology, Pathology

## Abstract

Osteoarthritis is the most common joint disease and a major cause of disability. Distinct biological processes are considered crucial for the development of osteoarthritis and are assumed to act in concert with additional risk factors to induce expression of the disease. In the classical weightbearing joints, one such risk factor is an *unfavourable biomechanical environment* about the joint. While the elbow has long been considered a non-weightbearing joint, it is now assumed that the tissues of the upper extremity may be stressed to similar levels as those of the lower limb, and that forces across the elbow are in fact very high when the joint is extended from a flexed position. This review examined the available basic science, preclinical and clinical evidence regarding the role of several *unfavourable biomechanical conditions* about the elbow on the development of osteoarthritis: post-traumatic changes, osteochondritis dissecans, instability or laxity and malalignment. Post-traumatic osteoarthritis following fractures is well recognized, however, the role of overload or repetitive microtrauma as risk factors for post-traumatic osteoarthritis is unclear. The natural course of untreated cartilage defects in general, and osteochondritis dissecans at the elbow in particular, remains incompletely understood to date. However, larger lesions and older age seem to be associated with more symptoms and radiographic changes in the long term. Instability seems to play a role, although the association between instability and osteoarthritis is not yet clearly defined. No data are available on the association of malalignment and osteoarthritis, but based on force estimations across the elbow joint, it seems reasonable to assume an association.

## Introduction

Osteoarthritis is the most common joint disease. It should be considered a heterogeneous group of syndromes affecting all joint tissues, although the articular cartilage and subchondral bone often show the most prominent changes [6]. The primary changes occur in the articular cartilage, followed by associated changes in the subchondral bone [13, 36]. More recently, the important and maybe even initiating role of the subchondral bone has been the focus of interest [23, 24, 37, 50].

Osteoarthritis results from the disruption of the balance between synthesis and degradation of extracellular matrix components by the chondrocyte in combination with increased uncompensated chondrocyte apoptosis [2, 13, 14, 23, 36]. Ageing profoundly alters chondrocyte function and matrix structure and function [27]. There is increasing evidence that cell senescence can result in phenotypical alteration of cells, called the *senescent secretory phenotype* [15, 16]. This phenotype is characterized by increased production of cytokines and growth factors. Accumulation of cells expressing this *senescent secretory phenotype* may contribute to tissue ageing, by stimulating matrix degradation and reducing matrix synthesis and repair, and possibly even directly link ageing to joint degeneration [36].

Amongst many, age has been shown to be the major independent risk factor for the development of osteoarthritis. Ageing and osteoarthritis are inter-related, not inter-dependent, cartilage is to some extent part of *normal ageing*. It is increasingly understood that ageing contributes to the development of osteoarthritis by working in conjunction with a variety of other factors, both *intrinsic* and *extrinsic* to the joint [36].

Osteoarthritis has traditionally been classified as *primary* (idiopathic, developing in previously undamaged joints in the absence of a clear causative mechanism or event) or *secondary* (caused by a well-recognized predisposing condition) [42]. With more and more aetiologic factors being recognized, the term *primary* osteoarthritis seems to reflect more the incomplete understanding of the etiopathogenesis than that it defines a specific form of osteoarthritis. More recently, a classification into three subsets of primary osteoarthritis (type I genetically determined, type II oestrogen hormone dependent, and type III ageing related), based on well recognized and important biological mechanisms, has been proposed [6]. These three distinct biological processes are considered crucial for the development of osteoarthritis and are presumed to act in concert with various risk factors to induce expression of the disease [6]. One such risk factor is a biomechanically unfavourable condition about the joint. At present, avoiding or correcting such unfavourable conditions are the only ways through which physicians can influence the development of osteoarthritis.

Normal synovial joints can withstand repetitive loading during normal activities for a lifetime without developing osteoarthritis [14, 49]. Mechanical demand that exceeds the capacity of the joint to repair itself plays an important role in the development and progression of joint degeneration [13, 14]. This *overloading* can take two forms. Excessive mechanical surface stress can directly damage articular cartilage and subchondral bone and adversely alter chondrocyte function [14]. Also, substantial micro-damage can result from impact levels far below the level needed to produce macroscopic fracture. This micro-damage may progress to detectable compromise of the articular cartilage. Loading rate [19] and shear stress [7, 8] are important variables.

Compared to osteoarthritis of the hip and knee, symptomatic osteoarthritis of the elbow seems is rare [17, 52], while radiographic degenerative changes are being noted much more frequently [45]. Rheumatoid arthritis is the most frequent form of osteoarthritis at the elbow, followed by post-traumatic arthritis. Men are four times more affected than women. The most common causative factor in primary osteoarthritis of the elbow seems to be related to microtraumata and to sports that put stress on the upper limbs, although studies of these associations have produced contradictory results [12, 28, 40]. Based on cadavers studies of the general population, it had always been assumed that elbow osteoarthritis starts at the radiocapitellar joint and from there progresses to the ulnohumeral joint [1, 25]. On the other hand, two recent image-based studies suggest that with symptomatic osteoarthritis the ulnohumeral joint is as much or more affected as the radiocapitellar joint [18, 35]. Potentially, the radiocapitellar compartment is affected first, while the ulnohumeral compartment is already involved when the degeneration becomes symptomatic.

In this review, the pathophysiological mechanisms by which biomechanical conditions about the elbow may result in osteoarthritis are discussed.

## Trauma and post-traumatic osteoarthritis

Post-traumatic osteoarthritis of the elbow following fractures is well recognized and primarily affects young males [38, 43]. The mechanisms responsible for the development of osteoarthritis following injury are complex and remain incompletely understood [27]. There seems to be an association between the development of post-traumatic osteoarthritis and the injury pattern and amount of energy absorbed within the joint [46]. Elbow fractures are often the result of a series of complex biomechanical events and therefore frequently involve associated (i.e. non-osseous) injuries [34]. Because these associated injuries and their consequences to the elbow joint may all contribute to the development of osteoarthritis, it is difficult to isolate the role of each individual injury or effect. The role of overload or repetitive microtrauma as risk factor for post-traumatic osteoarthritis of the elbow is not so clear. Surveys in Scotland revealed miners working at the coalface to have a higher prevalence of elbow osteoarthritis [5]. An association of sports-related exercise, in the absence of macroscopic trauma, and increased prevalence of elbow osteoarthritis has never been reported. In addition, radial head resection in case of ulnohumeral degeneration increases stress on the ulnohumeral compartment and is therefore suggested to lead to aggravation of the pre-existing degeneration [31, 51].

## Osteochondritis dissecans

**Fig. 1:**
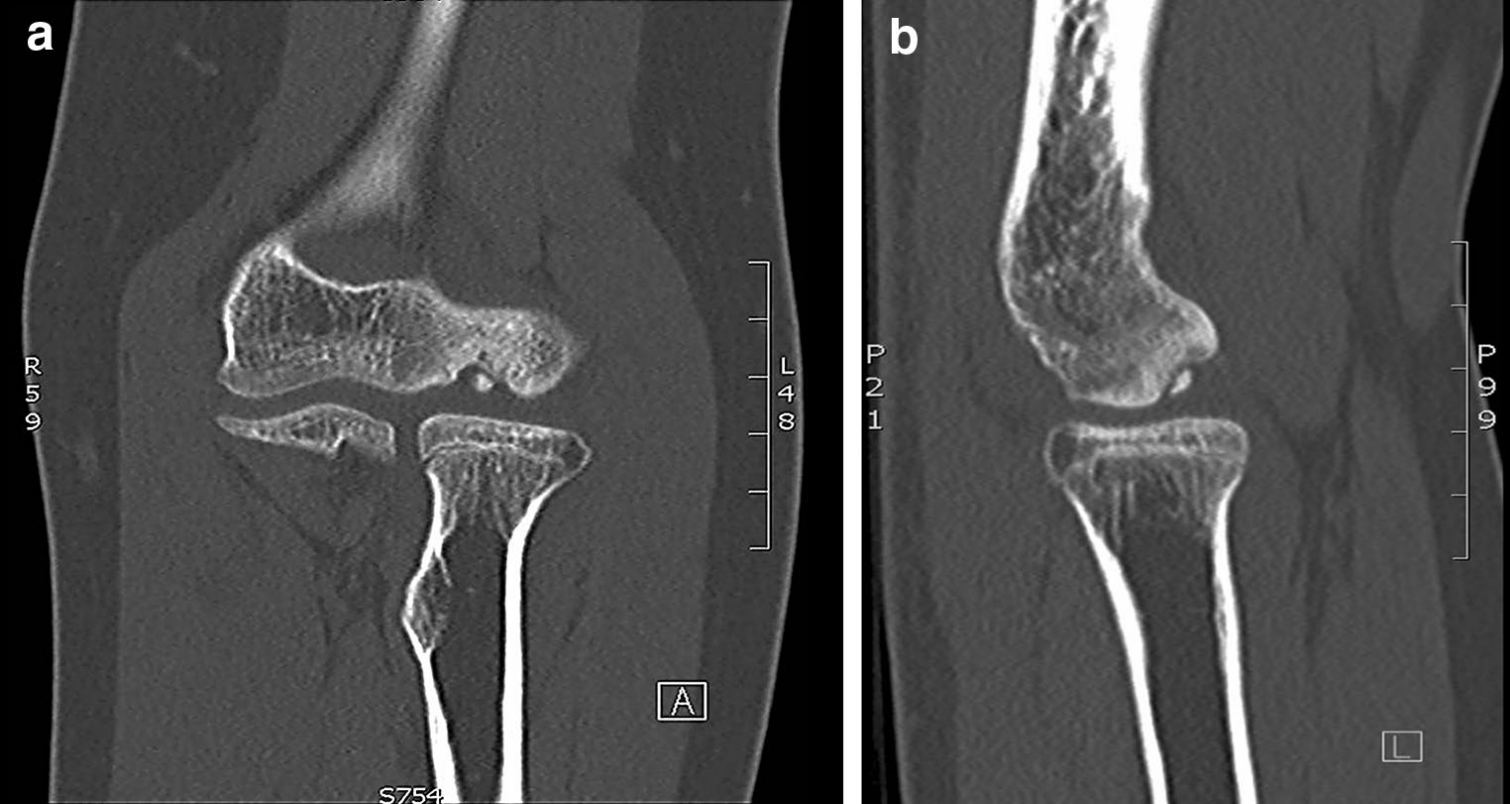
Coronal (**a**) and sagittal (**b**) CT-scan images of an osteochondral defect of the capitellum of the *right* elbow in a young girl with osteochondritis dissecans

Osteochondritis dissecans (Fig. 1a, b) is a process in which a segment of articular cartilage separates from the subchondral bone [44]. It is an uncommon disorder in the general population and presents typically in adolescent athletes engaged in repetitive overhead or upper extremity weightbearing activities (e.g. baseball, tennis, volleyball and gymnastics). The capitellum of the dominant elbow is most affected, however, bilateral involvement is seen in 20 % [48]. The aetiology is still unclear, but repetitive valgus forces across the elbow joint resulting in high compression loads at the lateral elbow compartment (‘valgus overload’) are thought to be the primary eliciting factor [9].

The relation to cartilage defects in general and the development of osteoarthritis in the long term is not clear to date. A large body of evidence is available about cartilage lesions in the knee [27]. With regards to the elbow, little is known about the cartilage lesions in osteochondritis dissecans and the risk of development of degenerative changes in the long term. In fact, even the natural course of untreated osteochondritis dissecans of the capitellum is still undefined to date [47]. Bauer et al. [10] observed a high incidence of elbow degeneration amongst 31 patients who had previously sustained osteochondritis dissecans at mean follow-up of 23 years. At end follow-up, 42 % of patients complained of pain and/or reduced range of motion. One-third had radiographic degenerative changes. It seemed that the younger the patient was diagnosed, the better the odds of having a pain free elbow with no radiographic signs of degeneration at end follow-up. The authors contributed this to better healing conditions at a younger age. Takahara et al. [47] noted a poorer long-term outcome of patients with larger cartilage lesions as compared to patients with smaller lesions. The authors concluded that this finding suggests that larger lesions may lead to degenerative changes over time and should therefore not be left untreated. However, no data are available whether any of the available cartilage defect repair strategies stop or slow down the development or progression of osteoarthritic changes at all.

In conclusion, the natural course of cartilage defects in general and untreated osteochondritis dissecans at the elbow in particular remain incompletely understood to date. However, larger lesions and older age seem associated with more symptoms and radiographic changes in the long term. No data are available whether any of the available cartilage defect repair strategies stop or slow down the development or progression of osteoarthritic changes at all.

## Instability

The elbow consists of a stable bony construct, surrounded by muscles and strong ligaments. The joint is stabilized by contraction of the muscles surrounding it. The passive ligamentous stabilizers will only be loaded when an external load overcomes the active stabilizing function of the muscles [3]. The ligaments of the elbow can be grossly divided into the *medial collateral ligament complex (MCL)* and *lateral collateral ligament complex (LCLC)*. The LCLC is assumed to be less important, because varus moments about the elbow are primarily resisted by the highly congruent osseous anatomy of the ulnotrochlear joint and because the elbow is mostly loaded in valgus due to the valgus carrying angle [41]. Somewhat simplified, three patterns of ligamentous injury are clinically recognized. The first is an injury to the medial collateral ligament caused by repetitive valgus stress due to overhead throwing type activities or axial compression. The medial collateral ligament can become attenuated over time or rupture, either acutely or following progressive weakening with attenuation. Secondly, instability can result from injury to the LCLC caused by forced external rotation of the elbow. Usually, this is a complete rupture of the ligamentous complex. The third type of instability is caused by simple dislocation of the elbow. Dislocations are mostly posterolateral in direction and lateral collateral ligamentous complex is always involved [21, 33].

A biomechanical study on cadavers by Eygendaal et al. [20] showed that complete rupture of the medial collateral ligament can result in an increase of 5.9 mm medial joint space opening during valgus stress with the elbow in 90° of flexion. The authors suggested that this would clinically result in damage of the articular cartilage of the radial head. A cadaveric study by Mullen demonstrated 50 % loss of valgus stability after sectioning of the medial collateral ligament [42]. This stability was almost fully recovered (97 % of initial stability with the elbow in 90° of flexion) after reconstruction of the anterior bundle of the medial collateral ligament. A cadaveric study by Jensen et al. [30] demonstrated that isolated reconstruction of the anterior bundle in the medial collateral ligament deficient elbow normalized joint varus-valgus and rotatory stability.

Only four clinical studies are available in the literature that focus on the association of instability and the development of osteoarthritis [11, 21, 32, 39]. A clinical follow-up study by Melhoff et al. [39] of 52 adults who had sustained a simple dislocation of the elbow and were treated conservatively showed no signs of radiographic degenerative changes at average follow-up of 34 months. A similar study by Boris et al. [11] looking at radiographic osteoarthritis after conservatively treated simple elbow dislocations in both children and adults, showed no degeneration in all 28 children at an average follow-up of 7 years. However, 11 out of 28 patients suffered from instability. In the adult group, radiographic osteoarthritis had developed in only one out of 34 adult patients at an average follow-up of 8 years, and eight out of 34 complained of instability. Josefsson et al. [32] observed radiographic degenerative changes or periarticular ossifications in 19 out of 52 patients (i.e. 37 %) at an average of 24 years follow-up after conservatively treated simple elbow dislocation. Similar observations were made by Eygendaal et al. [21] who noted radiographic degenerative changes in 21 out of 41 patients (i.e. 51 %) at an average follow-up of 9 years. In addition, 19 reported pain, eight had decreased flexion and 23 had a flexion contracture at end follow-up. They also noted evidence of medial instability on dynamic radiographic examination and found a statistical highly significant association between this medial instability and the development of osteoarthritis on MRI. The much lower incidence of osteoarthritis of the first two studies compared to the latter two could possibly be explained by the short duration of follow-up of the study by Melhoff et al. and the somewhat diffuse inclusion criteria of the study by Boris et al. There are no studies available investigating the effect of surgery on the development of elbow osteoarthritis at the long term.

In conclusion, elbow joint instability seems to play a role in the development of osteoarthritis in the long term, although the association between the two is not yet clearly defined. The effect of reconstructing elbow stability by ligamentous repair, augmentation or reconstruction on prevention of elbow osteoarthritis has never been investigated.

## Malalignment

**Fig. 2:**
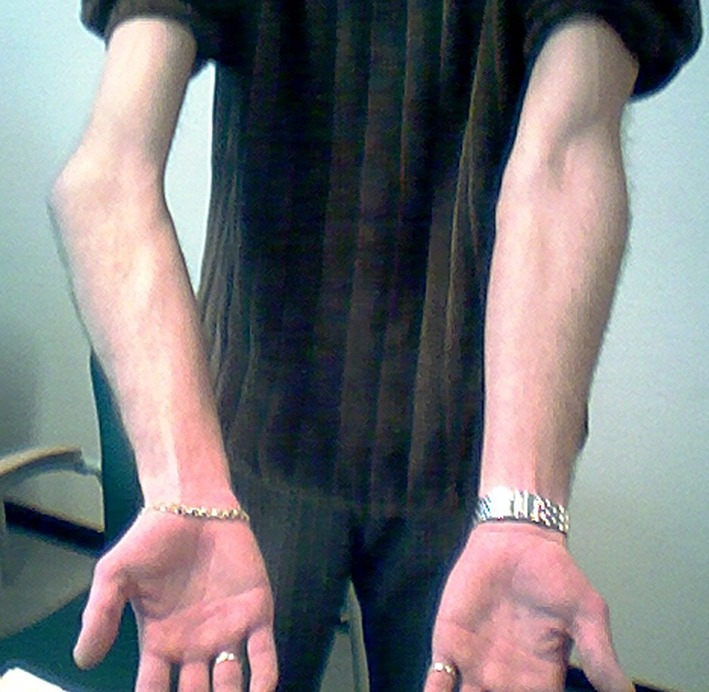
Post-traumatic varus deformity *(cubitus varus)* of the *left* elbow, posterior view

Malalignment of the elbow may result from malunion of intra- or extraarticular fractures, or from a combination of the two. Malunion with subsequent angular deformity of the elbow is mostly seen as an adverse sequela of supracondylar fractures in children. Varus deformity (*cubitus varus*) (Fig. 2) is more often reported than valgus deformity (*cubitus valgus*). In elbows with open growth plates, some remodelling can be expected, especially in the sagittal plane; no improvement is expected in the coronal plane or in case of rotational deformity [22].

The biomechanical consequences of malalignment of the upper limb relate to the distribution of forces transmitted from the distal humerus across the elbow joint to the forearm. The upper limb is often referred to as ‘non-weightbearing’. However, based on calculated loads across the normally aligned elbow joint and their effect on the relatively small bones and joint surface, it has been shown that the tissues are stressed to similar levels as those of the lower limb [29]. Forces across the elbow are in fact very large when the joint is extended from a flexed position due to the high forces needed to be generated by the triceps muscle to compensate for its small moment arm. As a result, the joint contact force at the ulnohumeral may be as much as twenty times as large as the external load acting on the hand and wrist [4]. No data are available on the effect of malalignment of the elbow on forces across the elbow joint. Despite the lack of evidence, it nevertheless seems reasonable to assume an association between malalignment and osteoarthritis, much alike the lower extremity [26].

In conclusion, no data are available on the effect of malalignment of the elbow on forces across the elbow joint. However, with the understanding that the tissues of the upper extremity are stressed to similar levels as those of the lower limb and that forces across the elbow are in fact very large when the joint is extended from a flexed position, it seems only reasonable to assume an association between malalignment and osteoarthritis.

## Conclusions

The available basic science, preclinical and clinical evidence regarding the role of several *unfavourable biomechanical conditions* about the elbow on the development of osteoarthritis of the elbow were examined. Post-traumatic osteoarthritis following fractures is well recognized and primarily affects young males. The role of overload or repetitive microtrauma as risk factor for post-traumatic osteoarthritis is unclear. The natural course of cartilage defects in general and untreated osteochondritis dissecans at the elbow in particular remain incompletely understood to date. Instability seems to play a role in the development of osteoarthritis of the elbow in the long term. No data are available on the effect of malalignment of the elbow on forces across the elbow joint. It is important to realize that many other factors may play a role in the development of osteoarthritis, some of which also via mechanically induced pathophysiological changes to the cartilage and subchondral bone.
